# Biodegradation of alachlor in liquid and soil cultures under variable carbon and nitrogen sources by bacterial consortium isolated from corn field soil

**DOI:** 10.1186/1735-2746-10-21

**Published:** 2013-03-01

**Authors:** Mansooreh Dehghani, Simin Nasseri, Zahra Zamanian

**Affiliations:** 1 Department of Environmental Health Engineering, School of Health and Nutrition, Shiraz University of Medical Sciences, Shiraz, Iran; 2 Department of Environmental Health Engineering, School of Public Health, and Center for Water Quality Research, Institute for Environmental Research, Tehran University of Medical Sciences, Tehran, Iran; 3 Department of Occupational Health Engineering, School of Health and Nutrition, Shiraz University of Medical Sciences, Shiraz, Iran

**Keywords:** Alachlor, Biodegradation, Carbon sources, Fars, Nitrogen sources, Mixed bacterial consortium

## Abstract

Alachlor, an aniline herbicide widely used in corn production, is frequently detected in water resources. The main objectives of this research were focused on isolating bacterial consortium capable of alachlor biodegradation, assessing the effects of carbon and nitrogen sources on alachlor biodegradation and evaluating the feasibility of using bacterial consortium in soil culture. Kavar corn field soil with a long history of alachlor application in Fars province of Iran has been explored for their potential of alachlor biodegradation. The influence of different carbon compounds (glucose, sodium citrate, sucrose, starch and the combination of these compounds), the effect of nitrogen sources (ammonium nitrate and urea) and different pH (5.5-8.5) on alachlor removal efficiency by the bacterial consortium in liquid culture were investigated. After a multi-step enrichment program 100 days of acclimation, a culture with the high capability of alachlor degradation was obtained (63%). Glucose and sodium citrate had the highest alachlor reduction rate (85%). Alachlor reduction rate increased more rapidly by the addition of ammonium nitrate (94%) compare to urea. Based on the data obtained in the present study, pH of 7.5 is optimal for alachlor biodegradation. After 30 days of incubation, the percent of alachlor reduction were significantly enhanced in the inoculated soils (74%) as compared to uninoculated control soils (17.67%) at the soil moisture content of 25%. In conclusion, bioaugmentation of soil with bacterial consortium may enhance the rate of alachlor degradation in a polluted soil.

## Introduction

Alachlor (2-cloro-N-(methoxymethyl)-N-(2,6-diethylphenyl)-acetamide) is an extremely toxic and highly mobile herbicide that is widely used for pre-emergence control of broad-leafed weeds and grasses in corn, soybeans and many other crops in Fars province of Iran. Alachlor is found in mixed formulations with atrazine, glyphosate, trifluralin, and imazaquin. The consumption rate of alachlor is 5 liters per hectare. It is a selective systemic herbicide, absorbed by germinating shoots and roots. It works by interfering with the plant's ability to produce protein and elongate roots [1].

Alachlor is a B-2 carcinogen [2] and a suspected endocrine disrupter [3]. Other than cancer, liver toxicity and eye lesions are the chronic toxic effects of this herbicide [4]. When directly applied to soil, it may leach into groundwater and thereby pose a potential health hazard to humans and animals [1]. Due to high water solubility of alachlor (242 mg/L), relatively low soil adsorption coefficient, alachlor can be detected in surface and groundwater and also in the finished drinking water of many countries [5,6]. It is regulated by USEPA with a maximum contaminant level (MCL) of 2 mg/L for drinking water [2]. The desirable maximum contaminant level goal (MCLG) has been set to zero with regard to drinking water standards.

The dissipation of alachlor in soil was found to follow the first-order kinetics. It is readily adsorbed on to soils with higher clay and organic matter [7,8]. Alachlor is relatively non persistent and degrade rapidly in soil [9]. Alachlor half-life is greatly influenced by soil type, temperature, soil pH and moisture. The major dissipation route for alachlor herbicide is biodegradation, runoff and leaching [10-12]. The rate and extent of biodegradation of chlorinated herbicides are reduced due to the adsorption and desorption efficiencies. Bioavailability of these contaminants is often the rate-limiting step in microbial degradation [13].

Bioaugmentation techniques have often involved the addition of acclimated indigenous microbes that can degrade the target compounds at accelerated rates. The microbes are isolated from the contaminated soil through the enrichment process. The enrichment culture technique develops a mixed culture able to degrade the target compounds [14] Biostimulation involves addition of nutrients that are deficient but required for biodegradation of a pollutant. The addition of nutrient causes an increase of microbial populations, thereby, increasing the number of indigenous microorganisms capable of degrading the pollutant [15]. Cometabolic biotransformation can be enhanced by an increase in microbial activity which is stimulated by the presence of the additional substrates [16].

Though alachlor is transformed in nature by many different processes including biotransformation, photochemical decay, precipitation and volatilization, biological transformations are believed to be most practically significant [17,18]. One study indicated that more than 90% of alachlor transformation was biotic [19]. Although many researchers showed that the degradation of alachlor is more rapid under aerobic condition, one study revealed that alachlor is transformed more rapidly under anaerobic conditions in aquatic systems using field-scale experimental units [17]. Felsot and Dzantor (1995) confirmed that several bacterial isolates have the ability to partially detoxified alachlor [15]. Alachlor-degrading *actinomycetes* have been isolated from soil and able to degrade alachlor in mineral salts medium [20]. Sette et al (2004) used *streptomycete* strains with the ability to degrade approximately 60-75% of the alachlor in 14 days [1]. An investigation showed that alachlor degradation by a selected microbial consortium was happened especially during the first several days of the incubation period. Atrazine degradation of more than 88% was achieved by bacterial consortium [14], complete disappearance of alachlor over the study time was not achieved [21]. A selected microbial consortium capable of degrading alachlor has been isolated from a contaminated soil from a 100-year-old mix-load site [22]. Chirnside et al. (2011) studied the biodegradation of aged residues of alachlor in a mix-load site soil by fungal enzymes. Thirty-two percent of atrazine and 54% of alachlor were degraded in the biometers using white rot fungus, *Phanerochaete chrysosporium*. The half-life for atrazine and alachlor was 8.0 and 3.0 days, respectively [23]. Ninety-nine percent of alachlor degradation was achieved in anoxic slurries of corn-cultivated soil due to the preexistence of microorganisms in the soil [24]. Candida xestobii has the ability to use metolachlor as a sole source of carbon for growth and also degraded 80% of alachlor after 41 h of growth [25].

Since Fars is an agricultural province of Iran and enjoys the top rank in wheat and corn production in the country in recent years, herbicides especially alachlor has been widely used as a selective herbicide to control broad-leaf and grassy weeds in agricultural corn fields. Therefore, the objectives of the study were to (i) enrich and isolate the bacterial consortium capable of alachlor biodegradation in the agricultural soil samples with a long history of exposure to alachlor, (ii) assess the effects of carbon and nitrogen sources on alachlor biodegradation rate and (iii) evaluate the feasibility of alachlor degradation by the bioaugmentation of the bacterial consortium in soil culture so that future insitu bioremediation strategies could be developed.

## Materials and methods

### Sampling and preparation

Soil samples for liquid cultures were taken from Kavar corn field with a long history of alachlor application (more than 10 years) in Fars province. The latitude and the longitude of Kavar are 29°11'N and 52°44'E, respectively. Soil samples for soil experiments were also taken from a field in Bajgah which has been under alfalfa cultivation for 3 years and has not received alachlor in the past 10 years. Soil samples were collected from 0–20 cm of soil depth with a hand-driven soil auger and stored at 4°C until they were used. The soil samples were air dried and passed through 2 mm sieve to be prepared for further microbiological examinations.

### Soil analysis

The general physiochemical characteristics of soil were determined. Hydrometer was used to determine soil textures using Guelph method. Other soil characteristics such as soil solution pH [26] and organic matter content [27] were determined. The soil texture in Kavar corn field was loam and the amount of sand, silt and clay distribution were in order of 47.44, 31.5 and 17.06%, respectively. Soil pH was 7.94. Organic matter content was 8.8 g/kg soil. The native soil characteristics at Kavar site was fine loamy, mixed, thermic Typic Haploxerepts. The soil texture in Bajgah field, was clay-loam and the amount of sand, silt and clay distribution were in order of 28.7, 33.3 and 32.0%, respectively. Soil pH was 7.5. Organic matter content was 17 g/kg soil. The native soil characteristics at Bajgah site was fine loamy, mixed, thermic, Typic Calcixerepts.

### Chemicals and analytical method

All chemicals were purchased from Merck (Germany). Alachlor standard was supplied by Accua Standard Europe, Switzerland. For alachlor detection a Shimadzu Model gas chromatography (GC-16) system with a 2 mm i.d column pack with 5% OV-17 Shimalide was calibrated and tested prior to injection of the samples. A Flame Ionization Detector was used to detect alachlor in the samples. The injector and detector temperature was set at 250°C and 300°C, respectively. The column temperature was maintained at 175°C for 18 min, and then increased to 190°C (at a rate of 5°C 1/min), hold at this temperature for 17 min, and then increased to 280°C at a rate of 5°C min^-1^, and finally hold unchanged for 2 min. The flow rate of nitrogen carrier gas, hydrogen, and air were set at 20 mL/min, 30 mL/min and 300 mL/min, respectively. The detection limit for alachlor was 0.01mg/ kg soil.

### Enrichment program

In order to isolate the bacterial consortium capable of growth on alachlor as a carbon source, the basal salt medium were prepared as described in Rousseaus [28]. Ten grams of wet soil was inoculated into 90 mL of the medium amended with 30 mg/L of alachlor, sodium citrate (2 mg/L), ammonium nitrate (100 mg/L) and delvocid (25 mg/L) after autoclaving. Delvocid was used to prevent the growth of fungi and pH was also adjusted to 7.0. The inoculated cultures were incubated aerobically on a reciprocal shaker (150 rpm) at room temperature in dark to preclude photolysis reactions. The culture was subcultured on the same medium at one-week interval. From the last step, a one-week-old culture, 10 mL was then transferred to 90 mL of freshly prepared alachlor medium for the period of 14 and 100 days. The remained alachlor after 10 days of the inoculation of liquid media was quantified by GC. Controls contained 1 g of sodium azide per liter as growth inhibitor. The bacterial consortium were harvested by centrifugation (6000 g for 20 minutes), washed twice with 0.1 mL phosphate buffer (pH=7.3).

### Effects of carbon and nitrogen sources on alachlor biodegradation

Different carbon compounds such as glucose (G), sodium citrate (SC), sucrose (SU), starch (ST) with three replications at a concentration of 2 g/L, and also the combination of these carbon sources such as G + SC, ST+SU, SU + SC, ST + SC, SU + G, and ST + G each at a total concentration of 2 g/L were added to alachlor mineral salt broth. After inoculation of the media supported with the carbon sources, they were incubated at room temperature and placed in dark. Control and blank without bacteria inoculation and no carbon sources, respectively, were also used. After 10 days, the residual of alachlor was measured.

To measure the influence of nitrogen sources on the efficiency of alachlor biodegradation by the mixed bacterial consortium in liquid culture, nitrogen sources as (ammonium nitrate and urea) routine fertilizers were added to alachlor mineral salt broth containing glucose and sodium citrate. Ammonium nitrate and urea as fertilizers were applied to corn field at a concentration of 600–825 and 200–400 kg/ha, the nitrogen percent for these fertilizers were 34 and 46%, respectively. Urea was added to alachlor minimal salt media at a concentration of 138–690 mg/L as N which was corresponded with 100–500 kg/ha. Ammonium nitrate was added to alachlor medium at a concentration of 170–306 mgL^-1^ as N which was corresponded with 500–900 kg/ha. After inoculation of the media supported with nitrogen sources, they were incubated at room temperature in dark for 10 days. Three replications were done for each nitrogen source. Control and blanks without bacterial inoculation and nitrogen source, respectively, were used for this study. After 10 days, the remained alachlor reduction without inoculation was less than 5%.

To measure the influence of pH on the efficiency of alachlor biodegradation by the bacterial consortium in liquid culture, different pH from 5.5-8.5 (interval of 0.5) with three replications was used in alachlor mineral salt broth containing glucose and sodium citrate and ammonium nitrate as a nitrogen source. Control without bacterial inoculation was also used.

### Effect of initial alachlor concentration and soil moisture on alachlor biodegradation in soil

Alachlor degradation rate by the bacterial consortium was measured in 100 mL capped Erlenmeyer flask containing soil samples. Ten grams of soil sample was brought to the desired soil moisture (7 and 25%) by the addition of sterile deionized water. The experimental design consisted of 36 flasks with 12 treatment and three replications for each treatment. Normal application rates for alachlor are 2–8 kg/ha (1–4 kg a. i. 1/ha). The initial alachlor concentration of 6.7 and 26.7 mg/kg soil are corresponded to 2 and 8 kg/ha, respectively. However, alachlor concentration of 53.3 mg/ kg soil is related to 16 kg/ha (8 kg a. i. 1/ha) which is considered a relatively high alachlor concentration and might have occurred due to an accidental spillage. After one day of incubation to allow herbicide sorption to the soil, inoculation with 300 μL phosphate buffer containing the mixed bacterial consortium was added to soil to yield 7.5×10^5^ bacterial cell 1/g soils as determined by plating on soil extract agar. A non-inoculated control received only a sterile phosphate buffer. The soil samples were mixed until they were homogeneously wet, and then incubated at room temperature in dark until the end of the experiment. Soil samples were extracted after 30 days of incubation. To extract alachlor from the soil, 30 mL of 99.8% methanol was added to the soil and shake for one hour. The mixtures were filtered and then collected organic phase volume was reduced in a rotary evaporator for about 15 min [29]. Then rinsed with 10 mL of 99.8% n-hexane and stored in a refrigerator prior to GC analysis. The recovery of alachlor from soil with this method of extraction was 95%. Soil moisture was maintained constant through the incubation by weighing and correcting for any weight loss by adding sterile deionized water. To maintain high population of alachlor degraders, the inoculation with the consortium was made every 5 days over a 30 days period for a total of 6 inoculations. Soil samples at the initial alachlor concentration of 6.7 mg/g soil were extracted at the incubation time of 2, 5, 10, 15, 20 and 30 days and analyzed to determine the remained alachlor concentration at each time intervals.

## Results

### Alachlor degrading-bacteria

Sixty-five bacterial strains were isolated from the bacterial consortium with a high capability of alachlor reduction rate. According to gram reaction, the bacterial strains were grouped into two types: gram positive (G+) and gram negative (G-). Fifty-three of isolates were (G-) and twelve of them were (G+). Oxidative-fermentative reaction showed that forty-seven isolates were aerobic bacteria and the rest (18 isolates) were facultative anaerobic.

### Alachlor reduction rate and effects of different parameters in liquid culture

**Figure 1:**
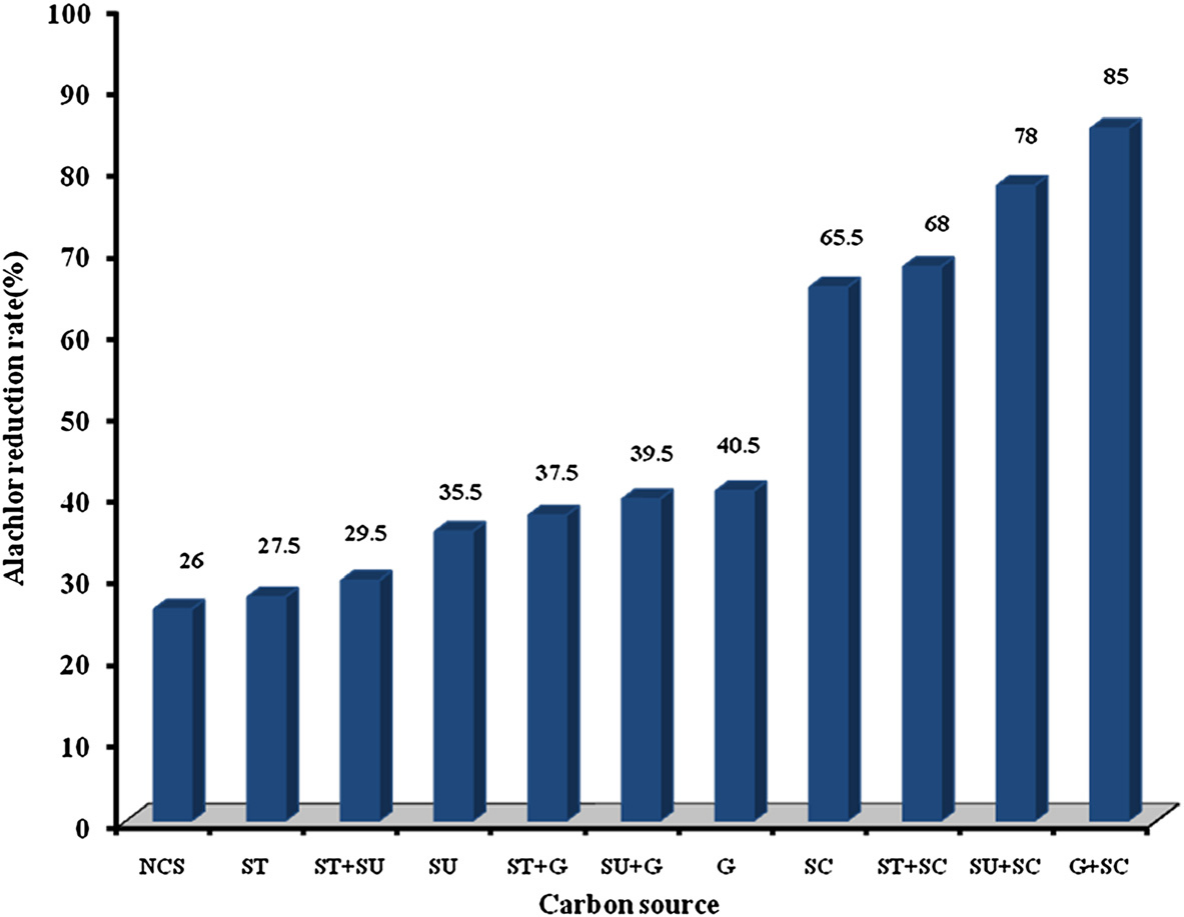
Alachlor reduction rate by the bacterial consortium at the different carbon sources in liquid culture.

In early enrichment culture, alachlor reduction was 20.0%. In late enrichment culture, its reduction was 63%. According to Figure 1, alachlor reduction rate for different carbon sources was in the range of 26 to 85%. Blank and control without carbon source and bacteria inoculation, respectively, were also used. A blank sample did not adequately support the growth of the consortium and the rate of alachlor degradation is less than 5%. Linear regression showed that there was a significant difference between various carbon sources and alachlor reduction rate (p<0.001).

**Figure 2:**
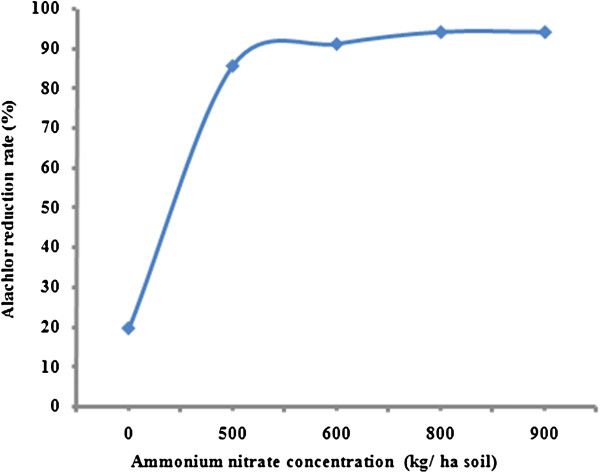
Alachlor reduction rate by the bacterial consortium at different ammonium nitrate concentrations in liquid culture.

**Figure 3:**
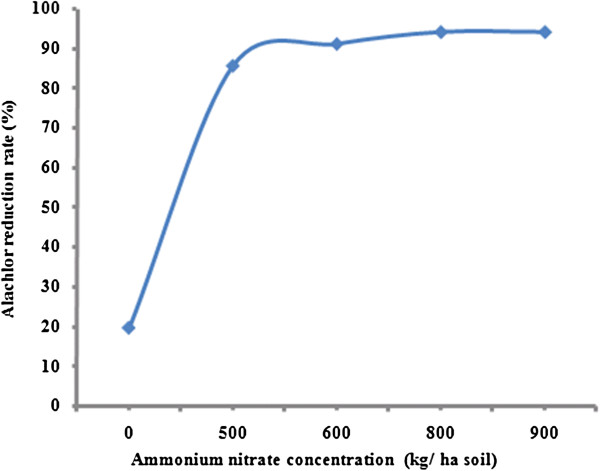
Alachlor reduction rate by the bacterial consortium at different urea concentrations in liquid culture.

The effect of different nitrogen sources on alachlor reduction were shown in Figures 2 and 3. Alachlor reduction rate increased from 19.5 to 94% as the ammonium nitrate concentration increased from 0.0 to 900 kg/ha soil. The rate of alachlor reduction for ammonium nitrate increased quickly when the concentration of ammonium nitrate increased from zero to 600 kg/ha soil, after that the degradation was getting slower. The same trend has been observed for alachlor biodegradation in the presence of urea. According to Figure 3, the percent of alachlor reduction rate increased from 19.50 to 88.50% as the urea concentration increased from 0.0 to 500 kg/ha soil. Alachlor reduction rate under nitrogen amendment showed an initial sharp increasing slope and then reaching constant with relative slower rate. Although linear regression analysis showed that a significant difference between the concentration of ammonium nitrate and alachlor reduction rate (p<0.05), there is no significant relationship between urea concentration and alachlor reduction rate was observed (p>0.05).

**Figure 4:**
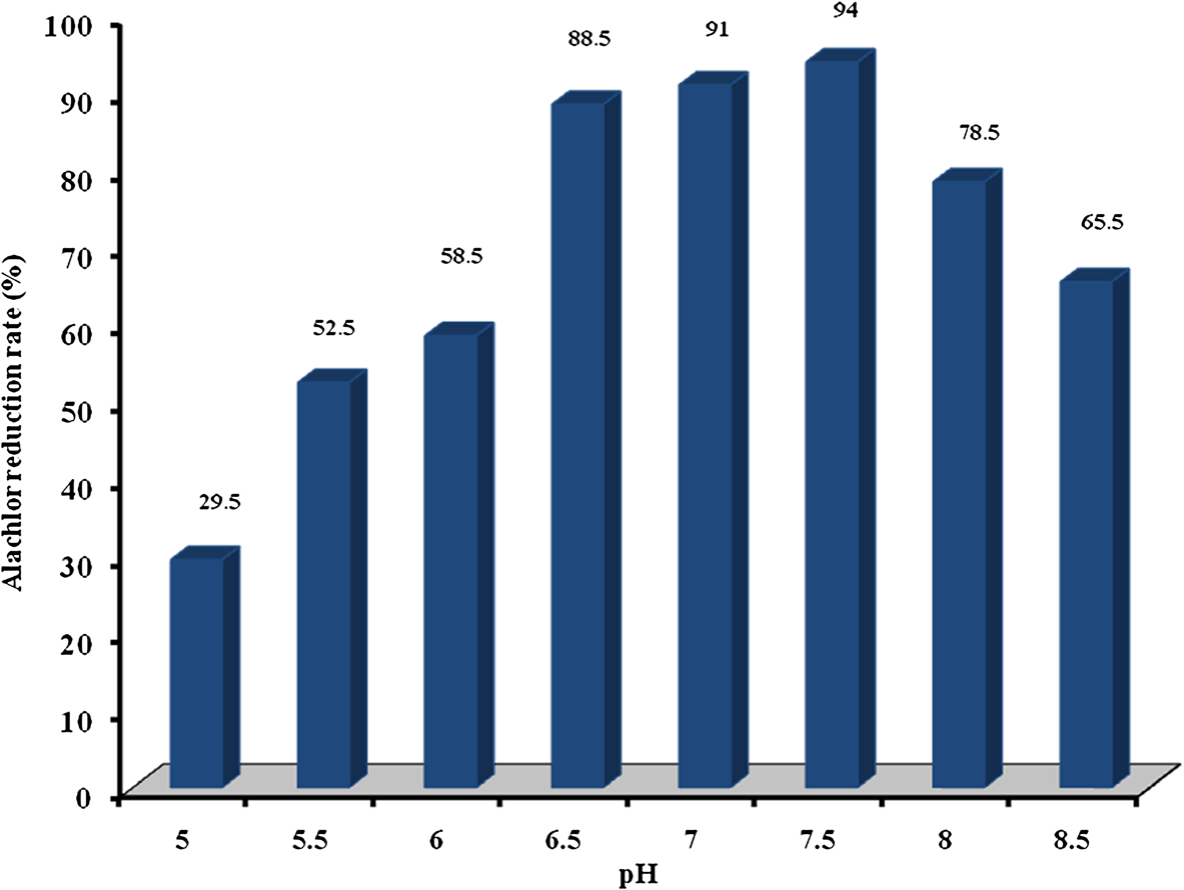
Alachlor reduction rate by the bacterial consortium at different pH in liquid culture.

The effects of pH on alachlor degradation rate were examined when glucose and sodium citrate were used as the carbon source and ammonium nitrate as the nitrogen source. The variations of pH on alachlor reduction rate were shown on Figure 4. According to regression analysis it can be concluded that there was a significant difference between pH and alachlor reduction rate (p<0.05).

### Effect of initial alachlor concentration and soil moisture on alachlor reduction rate in soil culture

**Figure 5:**
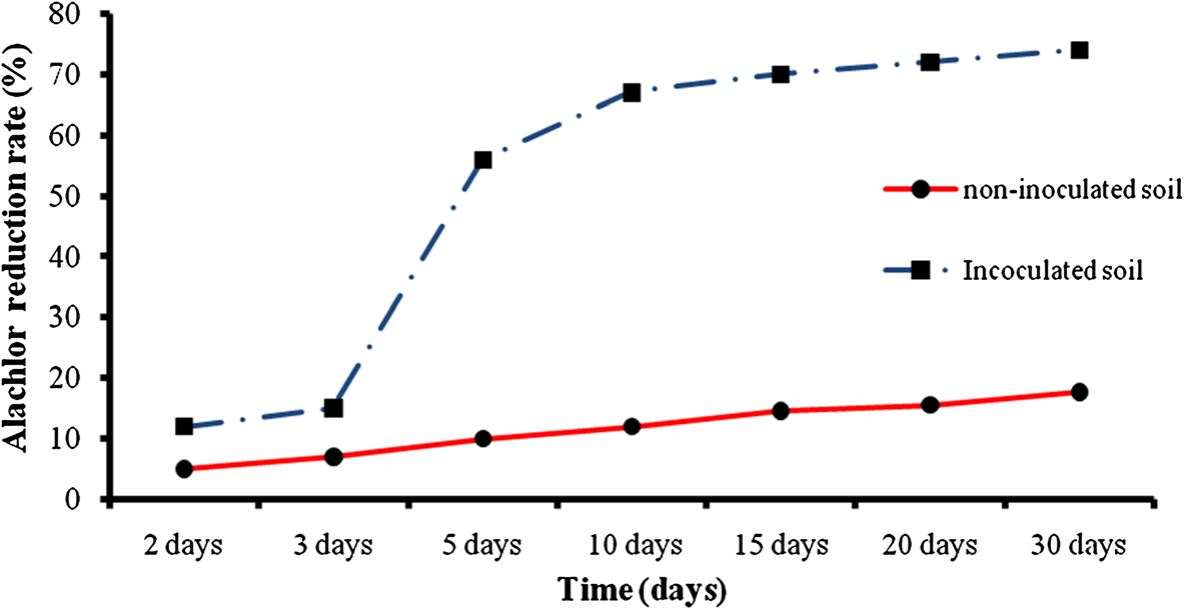
The percent of alachlor reduction rate for non inoculated and inoculated soil at 25% soil relative moisture content.

**Figure 6:**
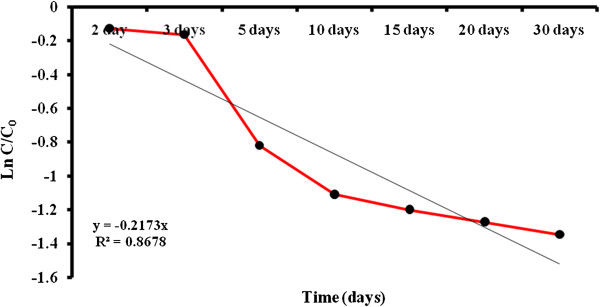
**Semi-logarithmic plot of concentration/initial concentration (C/C**_**0**_**) of biodegradation of alachlor by the bacterial consortium over time.**

Table 1 showed the effect of initial alachlor concentration and soil moisture content on the biodegradation of the inoculated soil. According to the data, after 30 days of incubation period, alachlor degradation rate for non-inoculated soil samples was low and alachlor reductions were only 9 to 17.67%. However for inoculated soil samples, degradation rate was higher and its reductions were 21.67-74%. For the inoculated soil samples at 25% soil moisture content at initial concentration of 6.7, 26.7, and 53.3 mg/g soil, alachlor reductions were 74, 65.67 and 33% within 30 days of incubation, respectively. Figure 5 showed alachlor reduction rate during the different time intervals in soil. Figure 6 depicted the plot of the semi-logarithm of initialized alachlor concentration (C/C0) versus time. During 30 days of incubation period, alachlor concentration decreased from an initial concentration of 6.7 mg/g soil to a final concentration of 1.74 mg/g soil. Only a 12% decrease in alachlor concentration was seen by the second day of the incubation period. During 12 days of incubation, 67% of the alachlor had degraded (Figure 5). After that a very slow increase in alachlor degradation was observed and remained almost constant until the end of the incubation period. The t_1/2_ for alachlor calculated from the plot was 7.16 d.

**Table 1:** The effect of inoculation of the bacterial consortium, initial alachlor concentration (mg/kg soil) and soil moisture (%) on alachlor reduction (%) after 30 days of incubation time in the soil culture

**Replication**	**Alachlor reduction rate, %**											
	**Inoculation (+)**						**Inoculated (−)**					
	**Initial alachlor concentration, (mg/kg soil)**											
	**6.7**		**26.70**		**53.3**		**6.7**		**26.70**		**53.3**	
	**Soil moisture, (%)**											
	**7**	**25**	**7**	**25**	**7**	**25**	**7**	**25**	**7**	**25**	**7**	**25**
**1**	35	69.5	29.0	62.5	21.5	34.5	16.0	19.5	12.5	14.5	10.0	11.5
**2**	34	78.5	31.0	68.0	23.0	31.5	14.5	18.0	13.5	16.0	9.5	10.5
**3**	38.5	74.0	33.5	66.5	20.5	33	10.5	15.5	10.0	12.0	7.5	12.0
**Mean**	35.83	74	31.17	65.67	21.67	33	13.67	17.67	12	14.16	9.0	11.33

## Discussion

Early enrichment culture indicated that the rate of alachlor degradation was not significant (20%) due to the toxic effect of alachlor on bacterial growth or the bacteria had not been able to use alachlor as a carbon source. Data regarding alachlor biodegradation showed that one successful enrichment culture at pH=7.0 was obtained with more than 60 percent of alachlor reduction. Since control media which were not inoculated and contained sodium azide as bacterial growth inhibitor, its reduction was less than 5%, it can be concluded that its reduction was not related to evaporation, photolysis and adsorption. Therefore, the decrease of alachlor concentration corresponded solely by the bacterial consortium. Generally, low alachlor level in the enrichment culture might reflect higher capability of alachlor degradation by bacterial consortium. Plating of bacteria on solid medium revealed the presence of many different colony types, suggested the combined metabolic activities of more than one bacterium. There is a more than a decade that alachlor and atrazine are both simultaneously used for the control of broad-leaf weed in Kavar corn fields and a study was done by Dehghani et al. also showed that atrazine biodegradation was enhanced in Kavar corn field soil compare to the other soils that had not been exposed to the herbicide [14]. The high degree of alachlor biodegradation appeared to be related to the site that had a long history of alachlor application due to the preexistence of microorganisms in the soil [21,24]. Many researchers found that the rapid alachlor biodegradation and mineralization was only observed when the microorganisms enriched by repeated subculturing or in soils inoculated with these adopted organisms [22]. Therefore, several application of alachlor on soil resulted in an enhancement of alachlor degradation.

Carbon sources of glucose and sodium citrate had the highest alachlor reduction rate. According to data, it is clear that sodium citrate had the major role in alachlor degradation. However, glucose as the only carbon source had resulted lower alachlor degradation compare to sodium citrate. According to data in this research, alachlor reduction rate with no carbon sources was only 26%. In laboratory conditions the carbon sources were added to support the higher growth of bacteria. Therefore, addition of carbon sources enhanced alachlor biodegradation due to cometabolism rather than direct metabolism. Past studies showed that the presence of additional substrates could initiate cometabolism of the desired compounds [15]. Bacteria and fungi can degrade alachlor through cometabolism [20,23,30].

According to data shown in Figures 2 and 3, cells grown on exogenous nitrogen source have increased alachlor reduction rate significantly. Alachlor utilization is activated under nitrogen sufficient condition and reduced under nitrogen limitation. Alachlor reduction rate increased more rapidly by the addition of ammonium nitrate compare to urea. Therefore, alachlor catabolism is significantly strengthened when additional nitrogen source is available. Many investigations showed a positive effect of nitrogen amendment on alachlor biodegradation by indigenous populations in soils [31,32]. Katz and his coworkers (2001) showed that *Pseudomonas* sp. strain ADP metabolized alachlor rapidly when cell grown on ammonium, nitrate, or urea [33]. Many researchers noted using mineral nitrogen greatly increased mineralization of alachlor in soil [31]. Organic nitrogen supplied in dairy manure increased alachlor mineralization. It can be concluded that the effect of nitrogen addition varies with the form and amount of added nitrogen.

Data regarding the effect of pH shows that as pH increased from 5.0 to 7.5, the rate of alachlor biodegradation increased. However, as pH increased from 7.5 to 8.5 caused a reduction rate in alachlor biodegradation. Based on the data obtained in the present study, pH of 7.5 is optimal for alachlor biodegradation. The reduction rate was more than 90% in this case. Dehghani et al. showed that the optimal pH for atrazine herbicide biodegradation was pH=7.0 [14].

Alachlor reduction rates were significantly enhanced in the inoculated soils as compared to uninoculated control soils. After 30 days, the percent of alachlor reduction was only 17.67% for uninoculated soils at initial alachlor concentration of 6.70 mg/g soil and the soil moisture of 25%. However, soil that was inoculated every 5 days with the bacterial consortium had reduced alachlor up to 74% at the same initial alachlor concentration and the soil moisture by Day 30 (Table 1). As initial alachlor concentration increased from 6.7 to 53.3 mg/g soil, alachlor reduction decreased from 74 to 33% (inoculated soils and soil moisture 25%). The decrease in alachlor reduction at 53.3 μg/g soil was possibly due to the result of complex interaction between microbial activity and nutrient availability. Therefore, unbalanced nutrient supply was probably responsible for the decrease. According to Table 1, enhanced alachlor reduction rates occurred with an increase in soil moisture from 7 to 25%. The percent of alachlor reduction increased from 35.83 to 74% as the soil moisture increased from 7 to 25% (at initial alachlor concentration of 6.7 mg/g soil). Many researchers found that higher herbicide reduction rate occurred with an increase in soil moisture. Soil moisture influences microbial processes through direct effect (e.g. water availability) or indirect effects such as solute diffusion, chemical availability and aeration [34]. Therefore the positive effect of increasing soil moisture was probably due to increased microbial mobility, solute diffusion and chemical availability which all had an indirect effect on alachlor degradation. The alachlor degradation reached a plateau at about 2 weeks. There are many reasons that degradation of alachlor became constant. One of the possibilities for the plateau is the shortage of necessary nutrients for growth of microbe and the other reason might be related to the nature of the alachlor. The bioavailability of a compound decreased with time until a minimum value is reached; at that point, no more degradation of alachlor will occur. Once the microorganisms degrade the available substrate present in the soil, the degradation rate is limited to the rate of desorption of the sorbed fraction back into the soil solution [35,36]. In this study, more than 70% of alachlor was degraded in 30 days of incubation periods (Figure 5). Assuming a pseudo-first order reaction for the disappearance of alachlor, a plot of the natural logarithm of initialized alachlor concentration (C/C_0_) versus time resulted in a rate constant equal to 0.0968 1/d (Figure 6). Alachlor degradation exhibited a short half-life of approximately 7.16 d. The initial slow degradation rate of alachlor was followed by a much faster degradation rate that lasted about Day 5 and then the degradation rate became slower and finally remained constant until the end of incubation period. Another study demonstrated that the half-life for alachlor was only 3.0 days. While the measured half-life in our study is about 2.5 times more than the Chirnside et al. study [23].

## Conclusion

In conclusion, our results showed that alachlor reduction rate was higher in liquid cultures than soil culture. The bacterial consortium in soil degraded 74% of alachlor at initial concentrations of 6.7 mg/g soil in 30 days. However, in liquid cultures, 94% of alachlor degraded in 10 days. Alachlor bioremediation in soil utilizing the bacterial consortium could be accomplished across a wide range of alachlor concentrations if the soil moisture were enough and also nutrient availability was balance too. Therefore, bioaugmentation of soil with the bacterial consortium with a high capability of alachlor biodegradation may enhance the rate of alachlor degradation in a highly polluted soil. It is very important to note that these bacteria in the controlled laboratory conditions (with the addition of carbon and nitrogen sources and without the interaction of environmental factors), had been able to utilize alachlor. However, there are some limitations to the use of acclimated microbial cultures to degrade the herbicide in a real field. Strains isolated in laboratory conditions might be stressed when reintroduced into the soil. Physicochemical conditions of soils and competition with native microorganisms may destroy or reduce the inoculums and limit its degradative capacity. Therefore, it is highly recommended that the feasibility of alachlor degradation to be studied by the bioaugmentation of mixed bacterial cultures to the real contaminated soil. The mixed bacterial consortium successful in laboratory studies may fail in the real field because of their sensitivity to high concentrations of other compounds.

## Competing interests

The authors declare that they have no competing interests.

## Authors’ contributions

The overall implementation of this study including design, experiments and data analysis, and manuscript preparation were the results of efforts by corresponding author. All authors have made extensive contribution into the review and finalization of this manuscript. All authors read and approved the final manuscript.
